# A Quality Improvement Initiative to Improve After-visit Summary Distribution in Orthopedic Outpatient Clinics

**DOI:** 10.1097/pq9.0000000000000620

**Published:** 2022-12-07

**Authors:** Jayme Williamson, Jessica Holstine, Julie Balch Samora

**Affiliations:** From the Nationwide Children’s Hospital Center for Clinical Excellence, Columbus, Ohio.

## Abstract

Communication and patient education are key to effective healthcare encounters and improved health outcomes. The after-visit summary (AVS) is an integral communication and education tool for patients and families. Without effective communication, gaps in comprehension may result in decreased satisfaction with medical encounters, lack of adherence to recommended treatment plans, missed appointments, and increased malpractice claims.

**Methods:**

We initiated a quality improvement (QI) initiative implementing concepts derived from the Institute for Healthcare Improvement (IHI) model, including plan-do-study-act cycles, to increase AVS distribution rates in a high-volume, fast-paced ambulatory pediatric orthopedic clinic. Interventions included staff education and training, trainee instruction, enlistment of electronic medical record superusers to enhance the distribution process, and provision of regular, transparent communication of individual and team performance. The impact of interventions was measured using a p-chart.

**Results:**

There was a consistent improvement in the rate of AVS distribution with each intervention implemented. The distribution rate on project initiation was 81.9%, with a final rate of 95.7%. The most statistically significant shift occurred following the final intervention, which included sharing unblinded individual performance data.

**Conclusion:**

Our data demonstrate that a dedicated QI program using IHI methodology improved AVS distribution rates in a pediatric orthopedic clinic. Consistently distributing the AVS affords our patients and families a better opportunity to review pertinent visit information, education, medication changes, and upcoming appointments.

## INTRODUCTION

Effective communication between healthcare providers and patients is associated with enhanced quality of care and patient satisfaction.^1^ Patients, or their caregivers, are often expected to retain details of medical information discussed during a clinic visit.^2^ Without effective communication, gaps in comprehension may result in decreased satisfaction with medical encounters, lack of adherence to recommended treatment plans, missed appointments, and increased malpractice claims.^2^ In addition, patient education interventions decrease hospitalizations, reduce visits to emergency departments, and improve patient quality of life.^3^

The after-visit summary (AVS) is a patient education and communication tool with relevant information regarding the clinic visit, including treatment regimens and upcoming appointment information. Patients have reported personalized free-text instructions as helpful and easy to understand.^4^ The Centers for Medicare and Medicaid Services (CMS) created financial incentives for healthcare organizations to improve the use of the AVS tool as part of the meaningful use requirement within the Affordable Care Act.^5^ The requirement included providing patients with an AVS with relevant and actionable information and instructions after each office visit.^6^ CMS challenged healthcare organizations to develop workflows and best practices to meet this provision.

The AVS is a tool to optimize clinical outcomes by ensuring thorough and clear communication with families. Unfortunately, the orthopedic clinics in our organization had an 81.9% distribution rate of the AVS in the 7-month baseline period with 19,240 visits, which was suboptimal in meeting the organizational expectation that all families be offered an AVS, requiring a concerted effort for improvement. This quality improvement (QI) initiative aims to increase the percentage of AVSs disseminated to patients at all orthopedic clinics from 81.9% to 95% by December 31, 2020, and to sustain for 6 months.

## METHODS

This project occurred at a large, urban pediatric medical center with a busy orthopedic service that conducts more than 40,000 clinic visits annually at eight locations. The orthopedic department includes 12 orthopedic surgeons, 13 advanced practice providers (APPs), and more than 60 residents rotating from three affiliated institutions. The surgeons and APPs function in teams, caring for patients independently and concurrently.

**Fig. 1.**
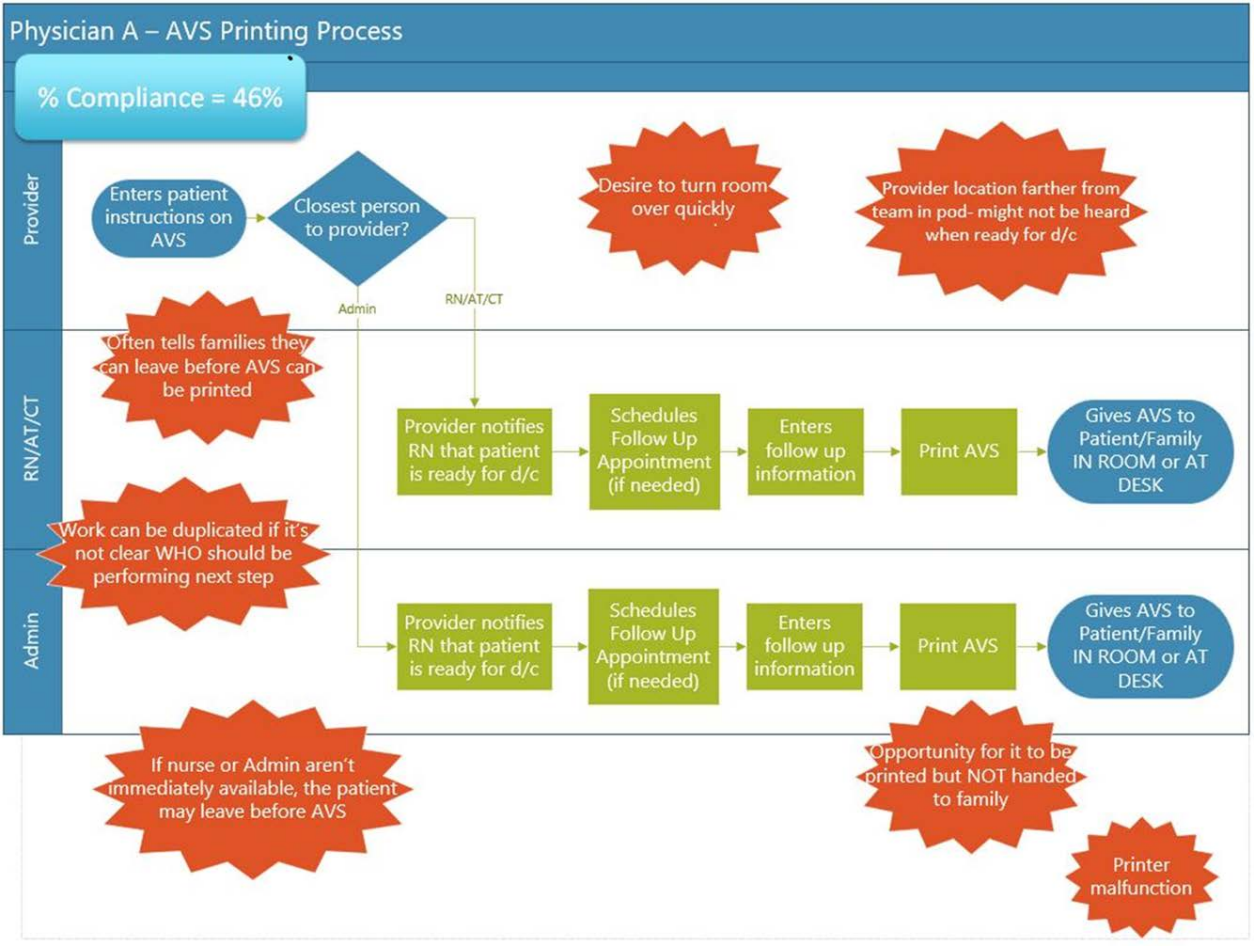
Process map that represents the most common clinic flow with swim lanes.

**Fig. 2.**
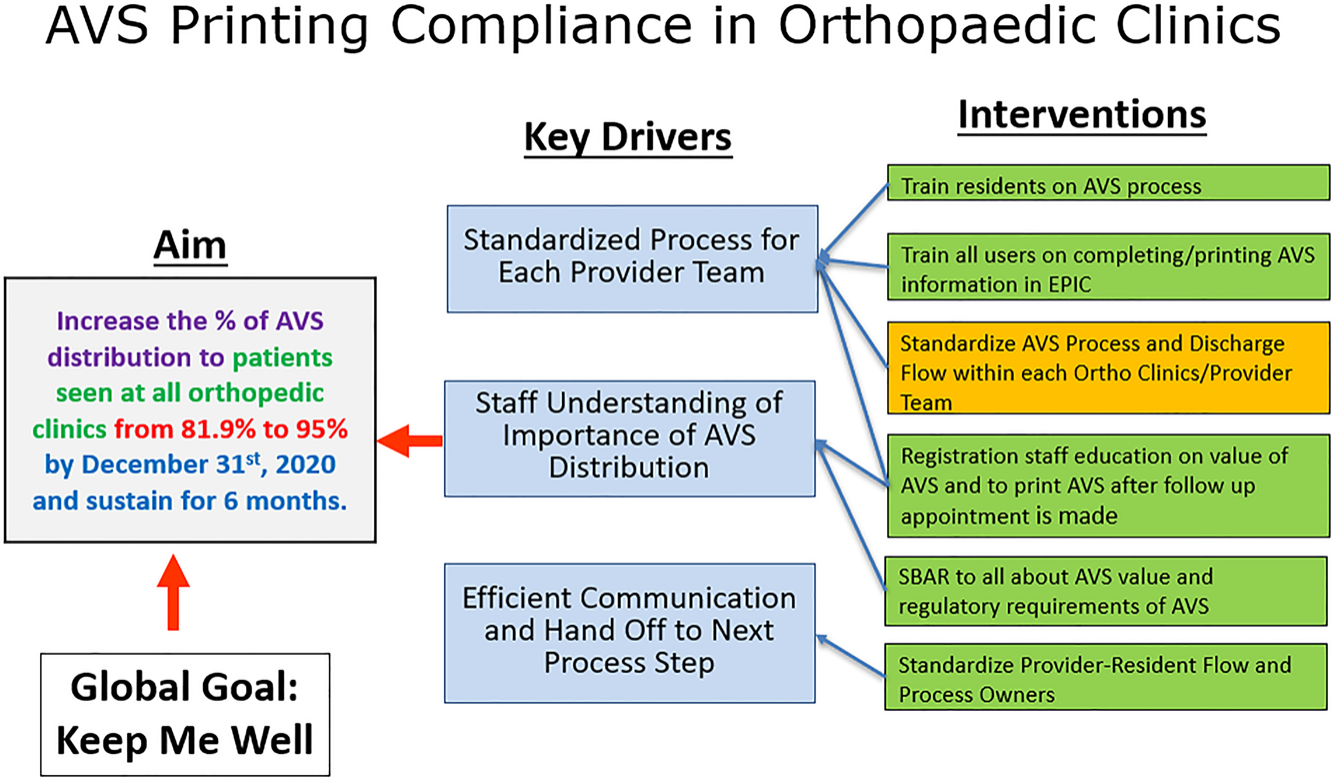
Key driver diagram for increasing the percentage of AVSs distributed to patients seen at all orthopedic clinics.

The QI project team included nurses, APPs, clinic leads, support staff, management, and QI professionals working in various locations. When reviewing baseline data to understand any special cause variation, provider teams exhibited high variability in their processes. Process mapping (Fig. 1), with current state swim lane maps for each provider, identified challenges to success. An affinity diagram determined the root causes of variability, leading to the key driver diagram (Fig. 2). QI work at Nationwide Children’s Hospital is exempt from IRB approval.

**Fig. 3.**
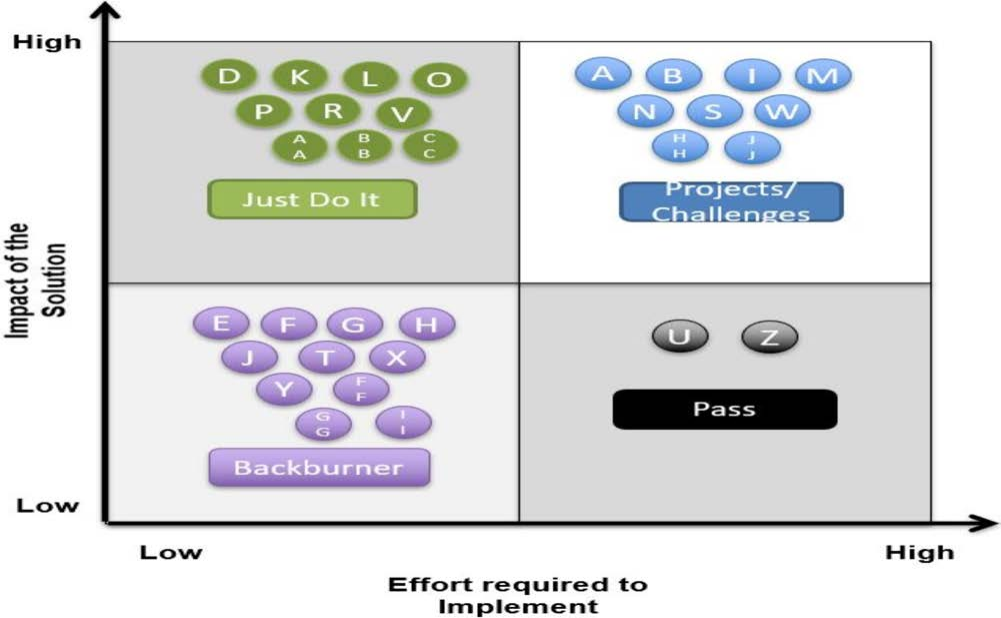
Impact/effort matrix used when evaluating value and ease of implementation of interventions.

A project group brainstorming session was conducted for interventions based on the key drivers and generated 53 ideas with 36 unique concepts (**Table 1, Supplemental Digital Content 1,** http://links.lww.com/PQ9/A442). An impact/effort matrix (Fig. 3) was used to evaluate each idea to assess its anticipated change management effort and potential positive impact on the overarching goal. The team prioritized interventions with high impact and low effort as “Just Do It” interventions. These interventions focused on raising awareness of the AVS distribution problem by filling in education and training gaps. We began with a department-wide communication, utilizing a Situation-Background-Assessment-Recommendation format (SBAR), informing all staff of the expectation that every patient receives an AVS after each visit.

**Table 1.** Brainstormed Interventions for AVS Distribution Improvement

ID	Intervention Description	Status	Impact/Effort Category
A	Provider to fill out all express lane information before telling patient and staff that patient is ready for discharge	Completed/implemented	Project/challenge
AA	Align priorities, getting patients’ AVS adds a miniscule amount of time to the appointment. Understand that patient satisfaction does not always correlate with a speedy visit	Completed/implemented	Project/challenge
B	Better communication between provider or resident to clinic staff for each patient	Completed/implemented	Project/challenge
BB	Achieve buy in from all orthopedic staff members	Completed/implemented	Just do it
CC	Provide unblinded feedback to providers and team	Completed/implemented	Just do it
D	Communication to entire department that each patient must receive an AVS before they leave the building	Completed/implemented	Just do it
E	Make sure printers are added and staff are aware of how to add to their profile so AVS can be printed	Not implemented after impact/effort matrix	Backburner
F	Keep toner cartridges in stock so that when they are low, they can be replaced	Not implemented after impact/effort matrix	Backburner
FF	Include contact information on AVS—reduce use of business cards	Determined to be out of scope	Backburner
G	Awareness of importance for families: Providers encourage patient to stop and ask for an AVS at the administrative desk before leaving	Not implemented after impact/effort matrix	Backburner
GG	MyChart utilization if equipment malfunction, AVS can be viewed by family	Not implemented after impact/effort matrix	Backburner
H	Registration to check chart to make sure patients received an AVS as they are leaving	Not implemented after impact/effort matrix	Backburner
HH	Education on dot phrases and smart phrases to increase the value of the AVS	Determined to be out of scope	Project/challenge
I	Encourage provider to start their express lane before going into room, so finishing the express lanes after leaving room is not as time consuming	Determined not needed based on improvement through other interventions	Project/challenge
II	Double-sided printing available on all printers, less paper is easier for families	Not implemented after impact/effort matrix	Backburner
J	Delineation of roles and standardized workflow. A general guideline for which job titles should be doing this so there is not confusion between teams	Determined to be out of scope	Project/challenge
JJ	Link helping hands to the AVS, combine documents or use helping hands to create dot phrases and/or smart phrases	Determined to be out of scope	Project/challenge
K	Train registration that when making a follow-up appointment, print AVS after so that appointment date and time are on AVS	Completed/implemented	Just do it
L	Train residents to communicate to staff when someone is ready for discharge, what their follow-up should be and do not send them out without letting the team know. Include in the standardized process in resident training	Completed/implemented	Just do it
M	Express lane completion before provider moving on to next patient is critical to having the correct information on the AVS	Determined not needed based on improvement through other interventions	Project/challenge
N	Provider flow standardization: All providers should have some sort of standardization with resident	Completed/implemented	Project/challenge
O	Help registration understand the AVS holds a lot of valuable information for families	Completed/implemented	Just do it
P	Fill training gaps related to AVS: Staff were not adequately trained specific to AVS when EMR launched	Completed/implemented	Just do it
R	Train all staff on how to print an AVS	Completed/implemented	Just do it
S	Standardize discharge process	Completed/implemented	Project/challenge
T	Improve value of AVS content: Create standard discharge smart phrase that makes discharge process more efficient. Tailor each smart phrase by diagnosis and share with all providers	Determined to be out of scope	Backburner
U	Always have a backup printer available in a clinic	Not implemented after impact/effort matrix	Pass
V	Standardize AVS review with the family upon discharge; General scripting for staff giving the AVS to families to explain the importance	Determined not needed based on improvement through other interventions	Project/challenge
W	Utilize dot system to improve communication so people are aware patient ready for discharge	Determined not needed based on improvement through other interventions	Project/challenge
X	Folders for AVS and all other paperwork for every patient that leaves. Families would be less likely to lose the information and would be a good way for staff to notice if someone is leaving without an AVS	Not implemented after impact/effort matrix	Backburner
Y	Work with IS to assure we all know what printer the AVS is printing to in a clinic	Not implemented after impact/effort matrix	Backburner
Z	Utilize paper versions of the AVS if there is equipment downtime	Not implemented after impact/effort matrix	Pass

Since several staff disciplines may interact with patients and families at the end of the visit, everyone working with patients was trained to print and distribute the AVS. Education gaps were attributed to the department recently moving from paper charts to a new electronic medical record (EMR) within the previous 18 months. Therefore, EMR superusers updated a training tool for orthopedic technicians and patient access representatives to print the AVS and document distribution.

Resident orientation did not previously include AVS expectations training or resident and fellow roles in this process. Therefore, the education coordinator made the clinic discharge process more comprehensive, focusing on AVS requirements. Residents and fellows are not responsible for discharge but are integral in documenting the plan and follow-up information provided on the AVS. In addition, all staff received an updated quick reference card that reflected the process.

**Fig. 4.**
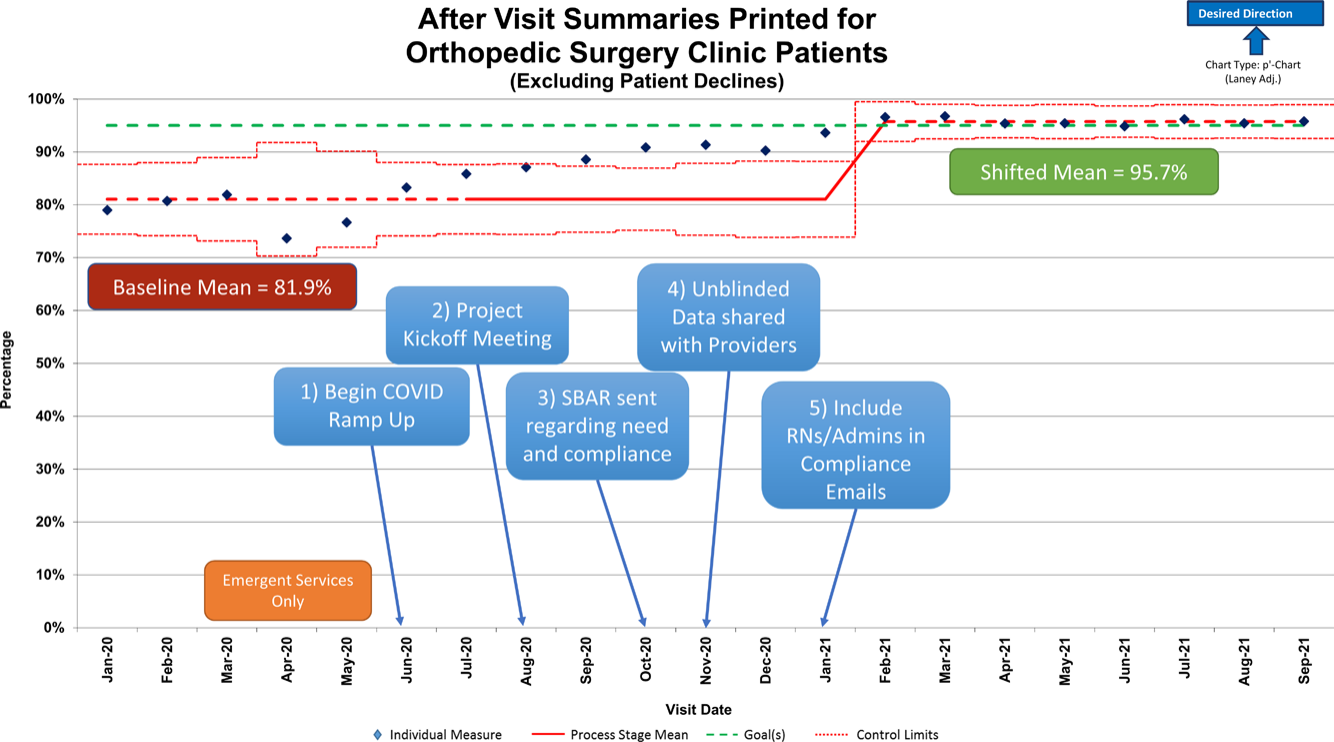
Percentage of AVSs summaries printed for orthopedic surgery clinic patients.

Two of the most impactful interventions centered around individual performance transparency. The surgeons and APPs received unblinded individual performance data in the first intervention. This unblinded information on individual performance compared to peers led to increased engagement and enhanced performance. Data were distributed monthly, with a clear positive impact on performance (Fig. 4). Second, since the nursing team and administrative assistants are generally the last disciplines to interact with the patient at the end of the visit, they often print and distribute the AVS. After 2 months of distributing the performance data to the surgeons and APPs, the RN and administrative team members were also included in the communications to raise performance awareness and buy-in, which proved to be another powerful intervention.

Weekly reports procured by the departmental data analyst directly from the EMR were used to measure improvements. Each intervention had a marked completion date and was reflected on the statistical process control p-chart with a Laney adjustment applied (Fig. 4) to evaluate the effectiveness of that intervention. The balancing measure for this project was appointment length. Although receiving the AVS improves the quality of the information provided, we wanted to ensure it was not at the expense of a significantly longer visit.

## RESULTS

Baseline data from January to July 2020 showed an average AVS distribution rate of 81.9% (n = 19,240). Patients who expressly declined the AVS were documented and removed from the data set. This project occurred during 2020–2021 when the COVID-19 pandemic increased family and staff avoidance of shared items. At the outset of the pandemic (April 2020), the distribution rate decreased significantly, briefly dropping below 75%. As additional information regarding COVID-19 transmission became available, the distribution rate returned to the prepandemic rate (Fig. 4).

As the project team was formed in August 2020, informal discussions regarding the project naturally raised awareness regarding AVS distribution (Fig. 4). In early October 2020, the project team disseminated a department-wide SBAR, which resulted in data points above the baseline mean. By mid-October 2020, all staff had completed training and education interventions without substantial improvements. The surgeons and APPs received their first unblinded individualized performance data at the end of October 2020. In addition to the department-wide SBAR, this intervention generated the most progress in AVS distribution percentages (Fig. 4).

In January 2021, the monthly data distribution included the RN and administrative assistant teams. Including these teams raised awareness of performance and helped achieve their buy-in, which led to ongoing improvements in the data. As a result, the process mean significantly shifted to 95.7% in February 2021, which was maintained for 8 months (Fig. 4).

## DISCUSSION

Within a little over 1 year, we have achieved and sustained significant improvements in AVS distribution rates. Unfortunately, there is a lack of literature regarding AVS distribution rates in an ambulatory setting. This study is the first designed and reported for a location within 18 months after migration from paper to electronic documentation.

On initiating this project, improvement was evident in individual data points due to team discussions about the problem and data analysis before any interventions were implemented. The Hawthorne effect,^7^ where people’s behavior changes because they know they are being observed, was attributed to this shift. The shift was not expected to be sustained without further intervention as people will unlikely have the stamina to over-perform for longer experiments.^8^

Staff, trainees, and attending physician education regarding the CMS regulatory requirements and communicating the importance of AVS distribution were critical to project success. The contents of the AVS, including patient-specific instructions, medication lists, and upcoming appointments, were specifically highlighted to staff to emphasize the importance. The education and training gaps were attributed to the recent migration from paper to electronic documentation. Additionally, increased staff turnover led to an influx of new staff. Therefore, the education and training interventions focused mainly on new team members, implementing changes to the onboarding education to emphasize understanding of the AVS distribution process.

Providing real-time, transparent performance data proved to be a great motivator. Once teams could visually appreciate their compliance with the goal, and compare their performance to their peers, AVS distribution rates noticeably increased. In addition, once the teams were in the habit of distributing the AVS and seamlessly incorporating the process, we sustained rates above the goal. Ultimately, there was no impact on the balancing measure of average appointment length.

Limitations of our study include the difficulty of comparing our results with other similar projects in the literature, as similar studies are lacking. Furthermore, this project was completed during the COVID-19 pandemic, which likely affected the implementation and outcomes. It is unclear if the project would have had the same trajectory without this external influence. An additional limitation was that we did not measure how the family perceived the value added by the received AVS. QI initiatives are greatly supported, encouraged, and resourced throughout our institution. Similar resources may not be available at other institutions. Finally, in a multimodal QI project, the specific intervention which accounted for the most improvement is unclear.

## CONCLUSION

In conclusion, we achieved the goal of 95% distribution of after-visit summaries in our orthopedic clinics and sustained success over eight months. In addition, we are confident these interventions are easily translatable to other ambulatory healthcare settings. Finally, improving communication avenues to educate patients and families may improve healthcare outcomes and satisfaction with their healthcare experience.

## DISCLOSURE

The authors have no financial interest to declare in relation to the content of this article.

## Supplementary Material


