# Supplementary material for: A Quality Improvement Initiative to Improve After-visit Summary Distribution in Orthopedic Outpatient Clinics
Source: Pediatr Qual Saf. 2022 Dec 7;7(6):e620. doi: 10.1097/pq9.0000000000000620 (PMC9742082; doi:10.1097/pq9.0000000000000620)
Supplement: Supplementary file 1 [file pqs-7-e620-s001.pdf]

Table 1			
**ID**	**Intervention Description**	**Status**	**Impact/Effort Category**
A	Provider to fill out all express lane information before telling patient and staff that patient is ready for discharge.	Completed/Implemented	Project/Challenge
AA	Align priorities, getting patients' AVS adds a miniscule amount of time to the appointment. Understand that patient satisfaction doesn't always correlate with a speedy visit.	Completed/Implemented	Project/Challenge
B	Better communication between provider or resident to clinic staff for each patient.	Completed/Implemented	Project/Challenge
BB	Achieve buy in from all orthopedic staff members.	Completed/Implemented	Just Do It
CC	Provide unblinded feedback to providers and team.	Completed/Implemented	Just Do It
D	Communication to entire department that each patient must receive an AVS before they leave the building.	Completed/Implemented	Just Do It
E	Make sure printers are added and staff are aware of how to add to their profile so AVS can be printed.	Not implemented after Impact/Effort matrix	Backburner
F	Keep toner cartridges in stock so that when they are low, they can be replaced.	Not implemented after Impact/Effort matrix	Backburner
FF	Include contact information on AVS – reduce use of business cards.	Determined to be out of scope.	Backburner
G	Awareness of importance for families: Providers encourage patient to stop and ask for an AVS at the administrative desk prior to leaving.	Not implemented after Impact/Effort matrix	Backburner

GG	MyChart utilization if equipment malfunction, AVS can be viewed by family.	Not implemented after Impact/Effort matrix.	Backburner
H	Registration to check chart to make sure patients received an AVS as they are leaving.	Not implemented after Impact/Effort matrix.	Backburner
HH	Education on dot phrases and smart phrases to increase the value of the AVS.	Determined to be out of scope.	Project/Challenge
I	Encourage provider to start their express lane prior to going into room, so finishing the express lanes after leaving room is not as time consuming.	Determined not needed based on improvement through other interventions.	Project/Challenge
II	Double sided printing available on all printers, less paper is easier for families.	Not implemented after Impact/Effort matrix.	Backburner
J	Delineation of roles and standardized workflow. A general guideline for which job titles should be doing this so there isn't confusion between teams.	Determined to be out of scope.	Project/Challenge
JJ	Link helping hands to the AVS, combine documents or use helping hands to create dot phrases and/or smart phrases.	Determined to be out of scope.	Project/Challenge
K	Train registration that when making a follow up appointment, print AVS after so that appointment date and time are on AVS.	Completed/Implemented	Just Do It
L	Train residents to communicate to staff when someone is ready for discharge, what their follow up should be and do not send them out without letting the team know. Include in standardized process in resident training.	Completed/Implemented	Just Do It

M	Express lane completion prior to provider moving on to next patient is critical to having the correct information on the AVS.	Determined not needed based on improvement through other interventions.	Project/Challenge
N	Provider flow standardization: All providers should have some sort of standardization with resident.	Completed/Implemented	Project/Challenge
O	Help registration understand the AVS holds a lot of valuable information for families.	Completed/Implemented	Just Do It
P	Fill training gaps related to AVS: Staff were not adequately trained specific to AVS when EMR launched.	Completed/Implemented	Just Do It
R	Train all staff on how to print an AVS.	Completed/Implemented	Just Do It
S	Standardize discharge process.	Completed/Implemented	Project/Challenge
T	Improve value of AVS content: Create standard discharge smart phrase that makes discharge process more efficient. Tailor each smart phrase by diagnosis and share with all providers.	Determined to be out of scope.	Backburner
U	Always have a backup printer available in clinic.	Not implemented after Impact/Effort matrix.	Pass
V	Standardize AVS review with the family upon discharge; General scripting for staff giving the AVS to families to explain the importance.	Determined not needed based on improvement through other interventions.	Project/Challenge
W	Utilize dot system to improve communication so people are aware patient ready for discharge.	Determined not needed based on improvement through other interventions.	Project/Challenge
X	Folders for AVS and all other paperwork for every patient that leaves. Families would be less likely to lose the information and would be a good way for staff to notice if	Not implemented after Impact/Effort matrix.	Backburner

	someone is leaving without an AVS.		
Y	Work with IS to assure we all know what printer the AVS is printing to in clinic.	Not implemented after Impact/Effort matrix.	Backburner
Z	Utilize paper versions of the AVS if there is equipment downtime.	Not implemented after Impact/Effort matrix.	Pass
